# Involvement of cerebrovascular abnormalities in the pathogenesis and progression of Alzheimer’s disease: an adrenergic approach

**DOI:** 10.18632/aging.203482

**Published:** 2021-09-03

**Authors:** Song Li, Che Wang, Zhen Wang, Jun Tan

**Affiliations:** 1 Liaoning Provincial Center for Clinical Research on Neurological Diseases, The First Affiliated Hospital, Dalian Medical University, Dalian 116021, China; 2 Liaoning Provincial Key Laboratory for Research on the Pathogenic Mechanisms of Neurological Diseases, The First Affiliated Hospital, Dalian Medical University, Dalian 116021, China; 3 Department of Pharmaceutical Chemistry, School of Chemistry and Chemical Engineering, Liaoning Normal University, Dalian 116029, China; 4 Department of Neurology, The First Affiliated Hospital of Wenzhou Medical University, Wenzhou 325000, China; 5 Key Laboratory of Endemic and Ethnic Diseases, Guizhou Medical University, Guiyang 550004, China

**Keywords:** Alzheimer's disease, adrenergic receptors, β-amyloid, cerebrovascular, tau

## Abstract

Alzheimer’s disease (AD), as the most common neurodegenerative disease in elder population, is pathologically characterized by β-amyloid (Aβ) plaques, neurofibrillary tangles composed of highly-phosphorylated tau protein and consequently progressive neurodegeneration. However, both Aβ and tau fails to cover the whole pathological process of AD, and most of the Aβ- or tau-based therapeutic strategies are all failed. Increasing lines of evidence from both clinical and preclinical studies have indicated that age-related cerebrovascular dysfunctions, including the changes in cerebrovascular microstructure, blood-brain barrier integrity, cerebrovascular reactivity and cerebral blood flow, accompany or even precede the development of AD-like pathologies. These findings may raise the possibility that cerebrovascular changes are likely pathogenic contributors to the onset and progression of AD. In this review, we provide an appraisal of the cerebrovascular alterations in AD and the relationship to cognitive impairment and AD pathologies. Moreover, the adrenergic mechanisms leading to cerebrovascular and AD pathologies were further discussed. The contributions of early cerebrovascular factors, especially through adrenergic mechanisms, should be considered and treasured in the diagnostic, preventative, and therapeutic approaches to address AD.

## INTRODUCTION

Alzheimer’s disease (AD) is the most common form of neurodegenerative disease in elder population worldwide. It is estimated that, by 2060, the number of AD patients in Americans age 65 and older may increase to 13.8 million from 6.2 million today [1]. AD is clinically characterized as cognitive decline and psychiatric manifestations. The pathological hallmarks of AD brain are the accumulation of extracellular β-amyloid (Aβ) (senile plaques) and the intracellular twisted strands of the hyper-phosphorylated tau protein (neurofibrillary tangles). These changes in the brain are accompanied by the neuronal damage. AD is a progressive neurodegenerative disorder that can start decades before the appearance of clinical symptoms. Although several pathological mechanisms of AD have been identified, no satisfactorily effective therapeutics has been developed. Recently, cerebrovascular dysfunctions, as a possible cause in the development and progression of sporadic AD, have gained increasing attention [2–4]. Increasing evidence has indicated the involvement of various alterations in cerebrovascular structure or functions, such as the cerebrovascular microstructure, blood-brain barrier (BBB) integrity, composition of neurovascular unit, cerebrovascular reactivity and cerebral blood flow (CBF), in AD pathophysiology and cognitive defects [5, 6]. Recent findings further highlighted the prevalence of cerebrovascular disorders in Down syndrome patients and added to a growing body of evidence implicating cerebrovascular abnormalities as a core feature of AD rather than a simple comorbidity [7]. Moreover, adrenergic system, including α/β adrenergic receptors and their downstream molecular signaling process, might serve as the key approach to modulate these cerebrovascular abnormalities and progressive neurodegeneration [8]. In this review, we summarized the interplay between cerebrovascular function and AD pathologies in both AD animal models and AD patients. Moreover, the underlying adrenergic mechanisms were further discussed to explore potential targets for future AD diagnosis and therapy.

## Cerebrovascular dysfunctions in AD

### Abnormal cerebrovascular microstructure in AD

**Figure 1:**
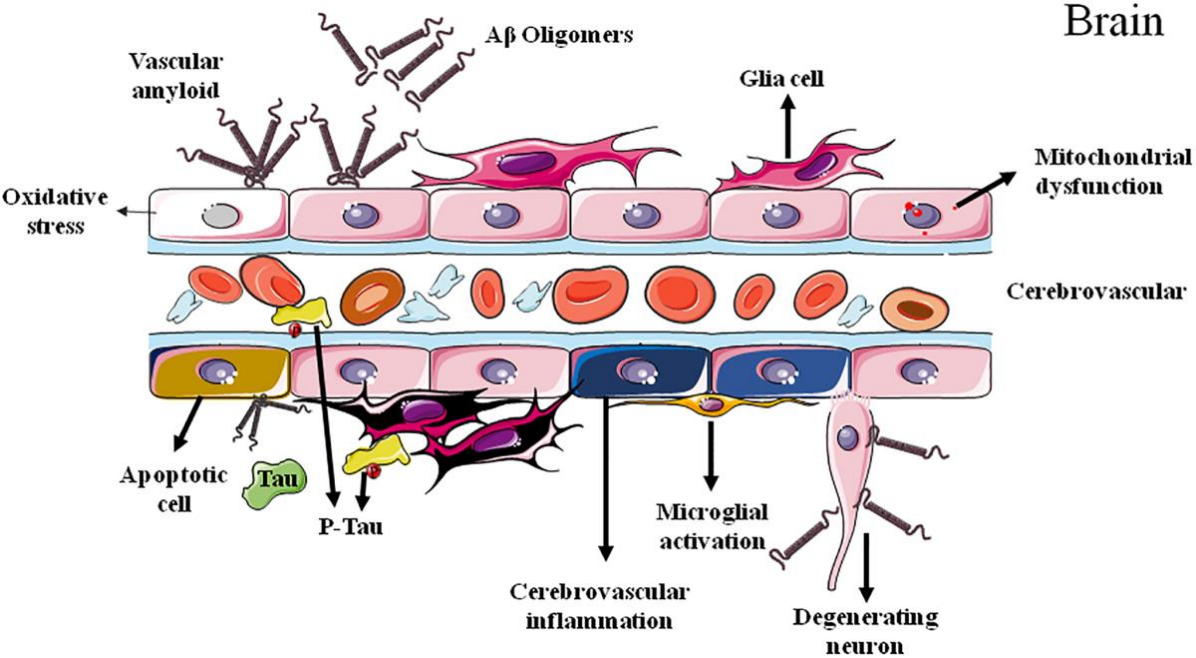
Cerebrovascular dysfunctions in Alzheimer's disease.

While a so-called ATN classification system of AD composed of Aβ [A] and tau [T] pathologies as well as neurodegeneration [N] have been established [9, 10], this system fails to present a clear causality and acknowledges an uncertain relationship between AD pathologies and AD symptoms. Considering the multifactorial feature of AD with complicate pathologies and manifestations, other factors, such as chronic and early cerebrovascular alterations, may contribute to the disease development and progression (Figure 1).

In 1991, microvessels in the brains of patients with AD were firstly observed by the scanning electron microscopy. Compared with control brain, obvious changes were observed in AD brains, including the focal constriction in terminal arterioles, irregular shape of smooth muscle cells, and the irregular abluminal surface and irregular constriction and dilatation of capillaries [11]. Microvascular ultrastructural changes in brain have also been reported to be prior to the onset of cognitive impairment in APP/PS1 AD model mice at 4 to 5 months of age [12]. Interestingly, despite the alterations of vasculature in brain, microvascular pathologies were also found in peripheral in AD [13]. At day 70 following femoral ligation, APP/PS1 mice showed significant decreases in cutaneous blood flow, collateral recruitment, capillary density and arteriole density in hind limbs compared to control wild-type littermates.

### Abnormal cerebral blood flow in AD

In the gray matter of AD brain, CBF can be reduced by more than 40%, with the largest decrease in the right supramarginal gyrus and the smallest in the right parahippocampal gyrus [14]. Moreover, in APP/PS1 and 5xFAD mouse models of AD, decreased CBF in cortical capillaries and impaired learning/memory ability was observed, which can be immediately ameliorated by the administration of antibodies against the neutrophil marker Ly6G [15]. The association between CBF alterations and AD pathologies can be observed in older adults without dementia. Non-demented individuals bearing high Aβ deposition in the brain showed declined CBF in right anterior to middle cingulate, right supramarginal gyrus, left thalamus, and midbrain bilaterally, as well as increase CBF in left medial and inferior frontal gyri, right precuneus, left inferior parietal lobule, and left postcentral gyrus, in the years preceding the neuroimaging acquisition [16]. Patients with amnestic MCI also showed a decreased cerebral perfusion [17] and this declined CBF in specific brain regions can be used as marker for identifying those MCI patients with high risk of rapidly progressing to AD in up to 3 years [17–21].

### Abnormal blood-brain barrier integrity in AD

Clinical and experimental studies have revealed disruptions of BBB integrity in AD [22, 23]. Much more interestingly, brain capillary damage and BBB breakdown in the hippocampus of individuals with early cognitive dysfunction were irrespective of the alterations of Aβ and/or tau pathologies, suggesting the promising potential as early biomarker for AD [24]. Additionally, pericytes maintain BBB integrity and clear Aβ from the brain, and their degeneration leads to BBB breakdown and accelerates the onset and progression of Aβ and tau pathology in mouse models of AD [25, 26]. Moreover, tissue from AD brains exhibited BBB leakiness and altered blood vessels morphologies, accompanied with amplified microglia reactivity and inflammation [27].

### Abnormal cerebrovascular reactivity in AD

Recent evidence suggests a close relationship between cerebrovascular function and AD. Cerebral hemodynamic alterations and decreased vascular density were found in the brain of APP/PS1 transgenic AD model mice [28]. Consistently, Hong et al. also found that, compare to wild-type control mice, APP/PS1 transgenic AD mice showed an increased cerebrovascular reactivity to ATP, which can be ameliorated by exercise training through P2Y2 receptor-mediated eNOS signaling and reducing ER stress [29]. Interestingly, the reactivity of large arteries was not affected in AD mice. Moreover, circulating Aβ might be responsible for the impaired peripheral vasculature repair mechanisms. These results were consistent with the Kelle et al. study, in which APP/PS1 transgenic mice showed a significant reduction of hepatic vessel number [12]. All these findings may suggest that vascular dysfunction in AD is a systemic alteration, both central and peripheral. Assessment of peripheral vasculature or vascular function may therefore provide additional tools for early diagnosis and management of AD.

### Cerebrovascular abnormalities as early sign of AD

Microvascular ultrastructural changes in brain of APP/PS1 AD model mice have been reported to precede cognitive impairment [12]. Despite those preclinical experimental findings in AD animal models, clinical studies of patients with AD also revealed early cerebrovascular dysfunctions prior to significant AD symptoms [30–33]. Moreover, brain capillary damage and BBB breakdown in the hippocampus of individuals with early cognitive dysfunction were irrespective of the alterations of Aβ and/or tau pathologies [24]. One recent cross-sectional study also provided Class II evidence that cerebrovascular reactivity in patients with cognitive impairment can predict cognitive performance independently of AD pathologic biomarkers [6], suggesting cerebrovascular abnormalities could be an early sign for AD.

Furthermore, during the very early stage of AD, PS1 mutation has been found to induce a decrease in cerebral perfusion in the hippocampal-amygdaloid complex and in the anterior and posterior cingulated cortex [34]. Similarly, individuals carrying high AD-risky apolipoprotein Eε4 (ApoE-ε4) allele showed a BBB breakdown and decreased CBF over time in the frontal, parietal and temporal cortices, preceding the occurrence of cognitive decline [35, 36]. These alterations were also independent of Aβ or tau pathology [36]. Therefore, CBF reductions in specific brain regions may be proposed as the early biomarker of the development and progression of AD [32, 33]. Much more interestingly, the higher basal level of soluble platelet-derived growth factor receptor β (PDGFRβ), one reliable BBB pericyte injury biomarker in the cerebrospinal fluid (CSF), may predict future cognitive impairment in APOE4 carriers but not in those non-carriers. These findings suggested that the abnormal structure or function of BBB contributes to APOE4-associated cognitive decline independent of AD pathology and might be a therapeutic target in AD patients carrying APOE4 [36].

## Interplays between AD pathologies and cerebrovascular alterations

As mentioned above, cerebrovascular abnormalities accompany and even precede the classical Aβ and tau hallmarks of AD, suggesting potential pathological interactions between cerebrovascular alterations and AD pathologies. In fact, polygenic risk scores for AD have been reported to be associated with various vascular events, including lobar cerebral microbleeds, white matter lesion load, and coronary artery calcification, suggesting vascular pathologies as an important target for future AD mechanistic research [37].

### Aβ-mediated vascular endothelial damage, blood-flow decline and vasoactivity

The Aβ precursor protein (APP) expression can be detected in vascular endothelium of both central and peripheral blood vessels. Expression and activity of the key enzymes responsible for both non-amyloidogenic and amyloidogenic processing of APP have also been detected in endothelial cells [38]. Intriguingly, expression of APP751 and APP770 isoforms is higher in endothelial cells of cerebral blood vessels than in those of peripheral arteries [39], predicting a more serious pathophysiological reactivity of cerebrovascular to Aβ.

CBF reduction has been recognized as an early sign of AD onset. Nortley et al. found that Aβ induced brain capillaries constriction at pericyte locations in biopsied human tissue of demented patients, via overproduction of reactive oxygen species (ROS) and subsequently the release of endothelin-1. Consistently, similar capillary constriction has also been observed in App^NL-G-F^ knock-in mouse model of AD [40]. Therefore, the Aβ-induced cerebral capillaries constriction may contribute to the energy lack and neurodegeneration in AD, and the inhibition of the capillary constriction caused by Aβ could potentially reduce pathological consequences of Aβ [40]. Further experimental evidence has specifically supported the deleterious impacts of Aβ on cerebrovascular function [41–43] (Table 1). For example, short-term incubation of arteries with Aβ increases the vasoconstrictions to phenylephrine, serotonin, and endothelin-1 [44–47]. Aβ accumulation, due to either elevated production or declined clearance, can induce arterial vasoconstriction, reduce CBF and lead to impairment of functional hyperemia [48–51]. It is worth noting that neurotoxic Aβ_42_ oligomer can also dose-dependently induced vessel disruption and reduced angiogenesis, which can be prevented by epidermal growth factor, suggesting angiogenic pathway as potential target for AD therapy [52].

**Table 1:** Effects of Aβ on isolated cerebral blood vessels [52].

**Vessel types**	**Aβ types**	**Concentration range**	**Cellular and/or molecular mechanisms of Aβ**	**References**
Rat cerebral artery	Aβ1–40	0.0001–1 μM	Impaired endothelium-dependent relaxation to ACh	[56]
	Aβ25–35	0.0001–1 μM		
Mouse cerebral artery	Aβ1–40	0.01–10 μM	Decreased cerebral blood flow to ACh	[58, 68]
	Aβ1–42	0.01–10 μM	No change observed	
Rat penetrating cerebral arterioles	Aβ1–40 Aβ1–42	0.001–1 μM	Reduction of tone diameter by increased contraction; decreased endothelium-dependent relaxation to ATP	[65]
Human middle cerebral artery	Aβ1–40	1 μM	Increased contractions to endothelin-1 via COX-2 and p38 mitogen-activated protein kinase activation	[61]
Human middle cerebral artery	Aβ1–40	2 μM	Increased production of PGF2α and PGE2	[61]
Bovine middle cerebral artery	Aβ1–40	1 μM	Impaired endothelium-dependent relaxation to bradykinin	[57]

Several possible molecular mechanisms have been identified to be responsible for Aβ-induced CBF reduction and cerebrovascular constriction, including the increased production of prostaglandins and ROS. Furthermore, incubation of cerebral arteries with Aβ impairs endothelium-dependent relaxations which can be reversed by free radical scavenger superoxide dismutase, supporting the involvement of increased ROS production and decreased eNOS activity in endothelial dysfunction [42, 44, 50, 53]. This vasoconstriction activity of Aβ might be also mediated by the altered function of the neurovascular unit (NVU) [54, 55] or α1 adrenergic receptor [47].

Despite the vascular tone, platelet aggregation also impacts the blood perfusion in brain. Platelets can attach to vascular amyloid depositions and thereafter initiate platelet activation, which induces hemostatic plug and vascular occlusion in the brain and leads to insufficient CBF. On the other hand, Aβ can bind to the integrin αIIbβ3 on platelets and stimulate the secretion of clusterin, which in turn promoted Aβ aggregation [56].

### Tau induces blood vessel abnormalities and cerebral blood flow changes

Despite Aβ, the pathological alterations of tau have also been reported to contribute to the cerebrovascular abnormalities in AD. Bennett et al. reported abnormal vascular morphologies, decreased vessel diameters and increased vessel densities in cortex in Tg4510 mice overexpressing P301L tau [57], which were concurrently accompanied by cortical atrophy and increased angiogenesis-related genes expression. Interestingly, all these cortical atrophy or blood vessel changes were not observed in Tg21221 mice overexpressing wild-type tau. Worth noting, Aβ accumulation in aged APP/PS1 mouse model of AD failed to induce plasminogen activation inhibitor 1 expression or similar blood vessel changes [58].

In addition, PS19 mice expressing mutant P301S tau and Tg4510 mice expressing P301L tau exhibit reduced vasodilatation of intracerebral arterioles and a suppressed CBF induction by neural activity, which precedes tau pathology and cognitive decline and can be reversed by reducing tau production [58]. All these vascular dysfunctions might be due to a tau-induced dissociation of neuronal nitric oxide synthase (nNOS) and reduction of nitric oxide (NO) during glutamatergic synaptic activity, indicating the glutamatergic signaling dysfunction and NO deficiency as early signs of tau pathology and providing a possible mechanism for the neurovascular alterations in the prodromal phase of AD [58].

### Cerebrovascular alterations induce and promote amyloidopathy in AD

The potential impacts of cerebrovascular abnormalities on amyloidopathy have been extensively investigated by detecting the susceptibility of developing AD pathologies under the reduced cerebral perfusion conditions in various animal models [59, 60]. Cerebral circulatory disturbance via the alterations of cerebrovascular structure or function may induce or exacerbate progressive neuronal loss in AD brain. Indeed, while intensive studies have focused on Aβ- and tau-related neuronal damages, increasing lines of evidence suggests the pivotal roles of vascular alterations in the pathogenesis and progression of AD [61]. According to the “Two-hit vascular hypothesis of AD”, the cerebrovascular damage preceding Aβ pathology is the initial insult of AD process. Reduced blood flow, BBB breakdown and altered vascular reactivity are self-sufficient to initiate neuronal injury and cognitive impairment (hit 1), and also subsequently alter Aβ production [60, 62, 63] and Aβ clearance [64] (hit 2). Faulty Aβ clearance itself may be the pathological consequence of arterial stiffness, which reduces the exchange of Aβ between interstitial fluid and CSF and leads to abnormal Aβ accumulation in brain [65]. ADAMTS13 deficiency led to an early and progressive damage of BBB together with reduced vessel density and decreased CBF in APP/PS1 model mice of AD. These cerebrovascular abnormalities induced by deficiency of ADAMTS13 also impeded Aβ clearance and consequently increased Aβ levels to exacerbate brain plaque load and cerebral amyloid angiopathy (CAA), resulting in worse cognitive decline in APP/PS1 mice. Interestingly, restoration of ADAMTS13 expression can attenuate BBB disruption, increase capillary perfusion, and ameliorate the CBF reduction in APP/PS1 mice. All these results confirmed the contribution of cerebrovascular alterations to Aβ pathology and further suggested ADAMTS13 as a novel target for AD therapy [66].

### Cerebrovascular alterations induce and promote tauopathy in AD

Despite Aβ pathology, the associations between vascular and tau and their effects on cognition have also been confirmed by voxelwise comparisons between CBF and tau PET images in independent discovery and replication cohorts. In participants with higher amyloid burden in brain and lower scores of Montreal cognitive assessment, strong correlation between soluble platelet-derived growth factor β and tau and correlation between CBF and tau were observed [67]. Laing et al. found that in AD patients, the volume of white matter of hyperintensity was positively correlated with plasma tau. Brain amyloid burden and the interaction between plasma tau and white matter of hyperintensity could distinguish AD and MCI patients from controls, with accuracies as 77.6% and 63.3%, respectively. Moreover, increased tau levels in plasma and CSF and hyperphosphorylated tau level in the ipsilateral hippocampus and cerebral hemisphere were observed in aged mice subjected to transient focal cerebral ischemia [68]. All these findings suggested cerebrovascular dysfunctions could induce and promote tau pathology in AD. Combination treatments targeting cerebrovascular, Aβ, and tau may be more effective to prevent or delay the pathogenesis or progression of AD than single-target therapies.

The chronic reduction of blood flow and thereafter impaired cerebral metabolism may deteriorate Aβ and tau pathologies through various molecular mechanisms. The Aβ/tau-neurovascular interactions are likely to fuel one another in deleterious vicious circles. Recently, Zhang et al. found that Aβ oligomers could be associated with α2A adrenergic receptor to induce glycogen synthase kinase 3β (GSK3β) activation and thereafter tau hyperphosphorylation [69], suggesting the possible involvement of adrenergic mechanism in cerebrovascular dysfunction and AD pathology.

## Adrenergic mechanisms underlying Aβ/tau-cerebrovascular interactions

The adrenergic signaling, as one of the major modulators of cerebrovascular reactivity and CBF, mediates its effects in blood vessels through various adrenergic receptors (also called adrenoceptors, ARs). ARs are a part of membrane-bound G-protein-coupled receptors (GPCRs) that mediate the peripheral and central actions of norepinephrine and epinephrine. Due to their distributions, either presynaptically or postsynaptically on neurons or effector organs such as the heart, vasculature, and adipose tissue, ARs mediate a broad range of important physiological homeostatic responses. ARs are classified into two main categories, α and β. The α type is further divided into α1 and α2 which are subdivided into α1A, α1B, α1D and α2A, α2B and α2C, respectively. β-ARs are classified into β1, β2 and β3 subgroups. Increasing evidence may predict the important roles of ARs and related signaling in the pathogenesis and progression of AD.

### α1-adrenoceptor

**Figure 2:**
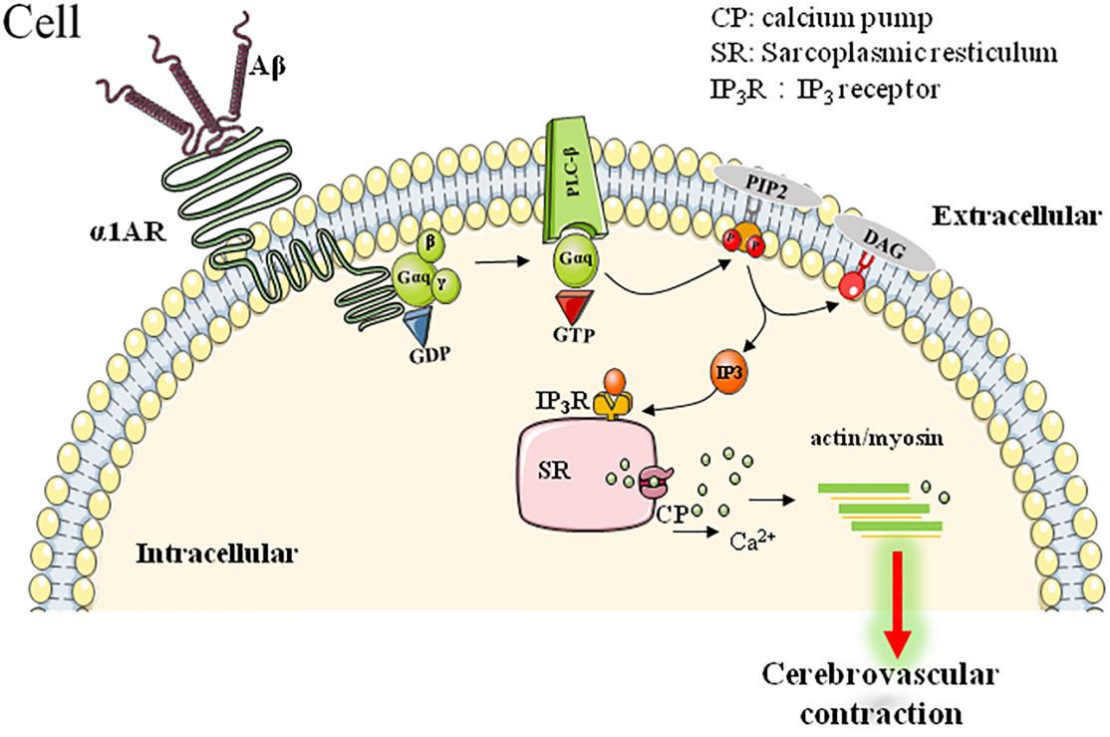
α1AR-mediated downstream signaling pathway involved in Aβ-induced cerebrovascular abnormalities.

The α1-AR is one of the ARs that play important roles in the regulation of vessel musculature. As a GPCR, the activated α1-AR couples with the Gq protein to activate phospholipase C, which cleaves phosphatidylinositol-4,5-bisphosphate into diacylglycerol and inositol trisphosphate, two second messenger molecules influencing cellular calcium homeostasis and activating PKC/ERK pathway to induce vasocontraction and cell proliferation (as summarized in Figure 2).

Increasing lines of evidence have been provided to support the deleterious roles of Aβ on cerebrovascular function [44]. Much more specifically, Haase et al. further found that Aβ25–35, the toxic fragment of Aβ, induced vasoconstriction of mouse aortic rings and coronary arteries in Langendoff-perfused rat hearts, which could be reversed by α1-AR blockage [47]. Consistently, agonistic autoantibodies (agAAB) for α1-AR have been found to exert similar impacts as Aβ did. α1-AR-agAABs were detected in about 50% of AD patients. These agAABs showed similar bioactivity as natural agonists do, such as Aβ, and bind to α1-AR to induce significant elevation of intracellular calcium. Moreover, these agAABs could cause vascular impairment in the brain of animal model, as shown by the significant CBF reductions and vessels damage. Interestingly, a subsequent 12-18 months follow-up observations found a stabilized cognitive performance while agAABs were removed by immunoadsorption in AD patients [70, 71]. All these data may predict the important roles of α1-AR-related vascular-pathologies in AD pathogenesis and progression.

### α2-adrenoceptor

Adrenaline or noradrenaline induce vasoconstriction through both α1- and α2-AR [72]. The α2-AR expresses in both endothelial cells and smooth muscle cells of vascular [73]. Interestingly, while the endothelial α2-AR induces vasorelaxation, the activation of α2-AR in vascular smooth muscle cells leads to vasoconstriction [74]. Although the signal transduction pathways behind α1-AR-mediated vasoconstriction have been well established, the exact mechanisms underlying α2-AR-mediated vasoconstriction are still largely unknown. α2-AR is coupled to Gi-protein and activation of α2-AR leads to a decreased intracellular cAMP level, which in turn results in constriction of vascular smooth muscle and an increased blood vessel tone. Moreover, cAMP/AC independent mechanisms have also been found to be involved in the α2-AR-mediated vasoconstriction. For example, α2-AR can induce the vasoconstriction in the porcine palmar lateral vein via the activation of ERK-dependent signaling pathway and Src tyrosine kinases [75]. Interestingly, compared to nondemented, low pathology control subjects, AD patients showed a significant increase of α2A-AR activity. Moreover, administration of α2A-AR activator clonidine resulted in worsened cognitive function in demented patients but not in non-demented normal subjects [69]. Although up to date no experimental evidence have revealed the direct binding of Aβ with vascular α2A-AR to modulate vascular function, Aβ oligomers but not monomer has been reported to interact with α2A-AR in α2A-AR overexpressed HEK293 cells. Moreover, Aβ oligomers can bind to allosteric site on α2A-AR to redirect norepinephrine-elicited signaling to GSK3β activation and so as to induce tau hyperphosphorylation [69]. Moreover, dexmedetomidine, an α2-AR selective agonist, induced a persistent hyperphosphorylation of tau in mouse hippocampus, which can be blocked by α2-AR specific antagonist atipamezole [76].

### β2-adrenoceptor

Despite α-AR, β-AR, especially β2-AR, is also an important target for modulating vascular tone. In general, acute β-AR activation induces vasodilation. However, chronic stimulation of β-AR led to an altered vascular responsibility to contractive insults. Chronic exposure of isoproterenol, a synthetic β adrenergic agonist, induced endothelial dysfunction and consequently an increased vasoconstrictive responsibility to phenylephrine [77]. The enhanced vasoconstrictor response and oxidative stress level induced by isoproterenol treatment might be due to the endothelial nitric oxide synthase (eNOS) uncoupling, through the downstream signaling pathway of β2-AR [78]. Previous studies have indicated that the stress-induced overproduction of catecholamines produce coronary spasms or arrhythmias via abnormal calcium signaling and impaired mitochondrial function. Notably, increased β2-AR expression level has been found in the brains of AD patients and β-blockers decrease the incidence of AD. In fact, several lines of evidence have supported the binding of Aβ with β-AR. Wang et al. revealed that Aβ has a binding capacity to β2-AR and induces PKA-dependent hyperactivity in AMPA receptors [79]. Aβ has been reported to induce tau phosphorylation at Ser-214, Ser-262 and Thr-181 through activating β2-AR-PKA-JNK signaling pathway in primary PFC neurons. Moreover, Aβ can also activate arrestin-ERK1/2 pathway in a β2-AR-dependent manner. In contrast, the Aβ-induced tau phosphorylation at Thr-181 can be partially reversed by inhibition of MEK, an upstream molecule of ERK1/2 [80]. All these findings indicated that Aβ could induce cerebral vascular dysfunction at least partially through β2-AR and its downstream signaling pathways. Much more importantly, activation of β2-AR could stimulate γ-secretase to promote the formation of amyloid plaque, which may predict a feed-back loop and vicious cycle to promote the progression of AD.

As for the correlation between β2-AR and tau pathology of AD, deletion of the β2-AR showed a reduced mortality and significantly improved motor deficits in tau transgenic mice. The improved lifespan and motor function was associated with a reduction in brain tau immunoreactivity and phosphorylation. The β2-AR-mediated changes in tau might be due to the reduced activity of GSK3β and cyclin-dependent kinase 5, two kinases responsible for tau phosphorylation [81].

### β1- and β3-adrenoceptors

Based on early pharmacological studies, the β2-AR was thought to be the major vascular responsive β-AR subtype, whereas β1 and β3 are primarily distributed in cardiomyocytes and adipocytes, respectively. However, experimental findings also reported that β1 and β3 ARs also play potential roles in the cerebrovasculature [82]. Previous studies found that chronic administration of CL316243, a specific β3 AR agonist, reversed the cognitive impairment and the elevated insoluble Aβ42/Aβ40 ratio in 16-month-old 3xTg-AD mice [83]. of CL316243 administration also specifically stimulated astrocytic but not neuronal glucose uptake and rescue the impaired cognitive function induced by Aβ [84]. As for β1-AR, chronic treatment with xamoterol, a selective, functionally biased, partial agonist of β1-AR, was effective to ameliorate cognitive deficits and reduce neuroinflammation and Aβ/tau pathologies in transgenic mouse models of AD [85, 86]. Furthermore, consistent with these findings, in APP transgenic mouse model of AD, chronic β-blockers administration potentiated CNS inflammation, whereas the β-blocker, metoprolol, induced phagocytosis and impaired cognitive behavior in both wild-type and APP transgenic mouse [87]. However, to date no direct evidence has implicated the involvement of β1- or β3-AR in the interactions of abnormal cerebrovascularure and Aβ/tau pathologies of AD.

## Clinical implications of cerebrovascular abnormality on AD early diagnosis and therapy

To-date, no clinically relevant therapies have been achieved addressing abnormal neurovascular functions of AD, aside from the reduced risk associated with diabetes and atherosclerosis. However, several clinical trials evaluated the therapeutic potential of adrenergic-agents against AD, including α2C AR antagonist ORM-12741 (https://clinicaltrials.gov/ct2/show/NCT01324518) and β2 AR agonist formoterol (https://clinicaltrials.gov/ct2/show/NCT02500784).

Several clinical trials have confirmed the benefits of α1-antagonist prazosin on AD. Several clinical trials have assessed the effectiveness of prazosin, one α1 AR antagonist, in AD. For examples, one randomized, placebo-controlled study in 24 patients suggested that prazosin significantly psychiatric symptoms of AD including agitation and aggression in AD patients, compared with the placebo-treated group. Another clinical trial found an improved behavior of AD patients after a 12-week period prazosin treatment compared with placebo control subjects. Moreover, another one synthetic α-AR blocker nicergoline also showed therapeutic potential against AD. Nicergoline has been found to enhance the cholinergic and catecholaminergic neurotransmission, improve the age-related cognitive deficits, and modulate protein kinase C (PKC)-mediated α-secretase processing of APP. Nicergoline has also been found to be neuroprotective and involved in the endogenous nerve growth factor-mediated processes. Nicergoline has been clinically used as therapeutics of cerebral metabolic-vascular disorders, vascular migraines, and dementia. In a European multicenter double-blind and placebo-controlled trial, nicergoline exerted a positive effect on the cognitive symptoms of mild-to-moderate AD [88].

### Retinal vascular changes as biomarker of AD

AD is pathologically characterized by progressive accumulation of Aβ, tau hyperphosphorylation, and progressive neurodegeneration. Pathological senile plaques and neurofibrillary tangles in brain have been accepted as hallmarks of AD. In addition, vascular changes occur at early stage of AD and are also involved in AD pathophysiology. Vascular changes may exacerbate Aβ pathology of AD through inhibiting Aβ clearance from the brain [89, 90], promoting influx of peripheral Aβ through the BBB [91], increasing APP expression [92, 93], which contribute to Aβ accumulation both in the parenchyma and blood vessels [94]. Consistently, altered BBB permeability and function precedes Aβ pathology and cognitive impairment of AD [95]. Therefore, assessment of vascular microstructure or function may provide valuable biomarker for early diagnosis of AD. Vascular signs may serve as novel biomarkers to provide pathophysiological insight for early diagnosis of AD. Although the currently used biomarkers for vascular changes are limited to MRI [96], recent research reports have indicated the possibility of using retinal capillary imaging as potential technique for AD diagnosis. For example, compare to wild-type mice, a significant retinal capillary degeneration in APP/PS1 mice was found as early as 8-month of age and was significantly worse during AD progression. This capillaries degeneration was correlated with substantial vascular PDGFRβ deficiency and prominent vascular Aβ deposition. Moreover, tight-junction alterations such as downregulated claudin-1 expression and increased permeability of brain-retina-barrier (BRB) were also found in APP/PS1 mice [97]. This retinal capillary degeneration and compromised BRB integrity at early stages in AD mouse may provide a new strategy for development of novel targets for AD diagnosis. Consistently, maximal venous and arterial reactions to monochromatic flicker stimulation were increased in AD patients as compared to healthy control and to subjects with MCI. Moreover, the arterial flicker response curve differed in MCI as compared to healthy control, as evidenced by significantly higher reactive magnitude and the more emphasized constriction in MCI [98]. These findings suggest that retinal structure or function is damaged during the early stage of AD. The retinal vessel reaction to flicker stimulation could be considered as a promising non-invasive and easy-to-administer biomarker for the diagnosis and monitoring of AD [98]. Similarly, Golzan et al. found that the amplitude of retinal venous pulsations was negatively correlated with the neocortical Aβ load, whereas the amplitude of retinal arterial pulsations was positively correlated with neocortical Aβ load. Retinal ganglion cell layer thickness was significantly decreased in AD patients. The correlation between retinal vascular alterations and Aβ load predicts a vascular component to AD pathogenesis and progression. Dynamic monitor of retinal vascular may provide reliable marker to aid in the assessment of AD during preclinical phase without significant cognitive manifestations [99]. Worth noting, one study in 2019, found that retinal vasculature did not discriminate AD patients from healthy controls [100]. However, much more recent experimental data were reported to support the utility of retinal vascular changes as potential marker of AD [75, 101–104].

### Cerebrovascular-targeting therapy against AD

While the current therapeutic strategies based on ATN theories yield very limited benefits against AD, recent studies have indicated that vascular lesions of dysfunction are involved in the pathogenesis and progression of AD. Various vascular events have been found to precede the pathological changes and clinical symptoms of AD, and vascular lesions share many common risk factors with AD. Vascular-recovering or remodeling may provide novel strategies to prevent the pathogenesis or delay progress of AD.

Microangiopathies have been found in APP/PS1 mice at 9 months of age, which can be ameliorated by administration of Liraglutide [105]. Consistently, implantation of mesenchymal stem cell-derived pericytes, the well-accepted modulator of cerebral vascular function, increased the brain microcirculation in APP/PS1 mice at the age of 18 to 20 months. Importantly, the levels of insoluble Aβ40 and Aβ42 together with the Aβ deposition were significantly reduced in hippocampus of the pericyte-injected hemisphere of APP/PS1 mice than that of the contralateral side. These findings indicated that the remodeling cerebral vascular via cell-based therapy can reduce brain Aβ-related pathologies, and could serve as a promising strategy for AD prevention and therapy [106].

Much more specifically, treatment of nicergoline, a selective α1A-AR blocker, may be beneficial to the severity of cognitive impairment, of daily living activities decline, and psychiatric symptoms in early AD patients. The follow-up study further revealed significant increases of relative regional CBF in AD patients. The increased perfusion in the superior frontal gyrus may be related to the delay or prevention of progressive cognitive deterioration in AD [107].

## CONCLUSIONS

AD is the most common cause of dementia, accounting for about 60% to 80% of cases. The pathological hallmarks including Aβ (plaques) and twisted strands of tau protein (tangles) are accompanied by progressive neurodegeneration and brain tissue damage. Unfortunately, so far, no therapeutic candidate, which inhibits Aβ aggregation or tau phosphorylation, has been approved to treat AD, and no satisfied early diagnostic scheme has been established for AD. The changes in CBF may impair cognition via promoting Aβ and tau pathologies or directly inducing neurodegeneration. Abnormal adrenergic activities such as AR-signaling may be involved in this process. Increasing lines of evidence from either preclinical or clinical studies have revealed that the cerebral vascular alterations during early stages of AD may contribute to the pathogenesis and progression of the disease. Cerebral vascular assessment may provide promising tools for AD early diagnosis and cerebral vascular remodeling may yield benefits to AD therapy.
